# Combining Metabolic Engineering and Multiplexed Screening Methods for 3-Hydroxypropionic Acid Production in *Pichia pastoris*

**DOI:** 10.3389/fbioe.2022.942304

**Published:** 2022-07-22

**Authors:** Albert Fina, Stephanie Heux, Joan Albiol, Pau Ferrer

**Affiliations:** ^1^ Department of Chemical, Biological and Environmental Engineering, Universitat Autònoma de Barcelona, Bellaterra (Cerdanyola del Vallès), Catalonia, Spain; ^2^ TBI, Université de Toulouse, CNRS, INRAE, INSA, Toulouse, France

**Keywords:** 3-hydroxypropionic acid, *Pichia pastoris*, glycerol, malonyl-CoA, acetyl-CoA, metabolic engineering

## Abstract

Production of 3-hydroxypropionic acid (3-HP) in *Pichia pastoris* (*syn. Komagataella phaffii*) *via* the malonyl-CoA pathway has been recently demonstrated using glycerol as a carbon source, but the reported metrics were not commercially relevant. The flux through the heterologous pathway from malonyl-CoA to 3-HP was hypothesized as the main bottleneck. In the present study, different metabolic engineering approaches have been combined to improve the productivity of the original 3-HP producing strains. To do so, an additional copy of the gene encoding for the potential rate-limiting step of the pathway, i.e., the C-terminal domain of the malonyl-CoA reductase, was introduced. In addition, a variant of the endogenous acetyl-CoA carboxylase (*ACC1* ^*S1132A*^) was overexpressed with the aim to increase the delivery of malonyl-CoA. Furthermore, the genes encoding for the pyruvate decarboxylase, aldehyde dehydrogenase and acetyl-CoA synthase, respectively, were overexpressed to enhance conversion of pyruvate into cytosolic acetyl-CoA, and the main gene responsible for the production of the by-product D-arabitol was deleted. Three different screening conditions were used to classify the performance of the different strains: 24-deep-well plates batch cultures, small-scale cultures in falcon tubes using FeedBeads® (i.e., slow release of glycerol over time), and mini bioreactor batch cultures. The best two strains from the FeedBeads® screening, PpHP8 and PpHP18, were tested in bioreactor fed-batch cultures using a pre-fixed exponentially increasing feeding rate. The strain PpHP18 produced up to 37.05 g L^−1^ of 3-HP at 0.712 g L^−1^ h^−1^ with a final product yield on glycerol of 0.194 Cmol^−1^ in fed-batch cultures. Remarkably, PpHP18 did not rank among the 2-top producer strains in small scale batch cultivations in deep-well plates and mini bioreactors, highlighting the importance of multiplexed screening conditions for adequate assessment of metabolic engineering strategies. These results represent a 50% increase in the product yield and final concentration, as well as over 30% increase in volumetric productivity compared to the previously obtained metrics for *P. pastoris*. Overall, the combination of glycerol as carbon source and a metabolically engineered *P. pastoris* strain resulted in the highest 3-HP concentration and productivity reported so far in yeast.

## Introduction

Microbial production of platform chemicals is an increasingly attractive alternative to petrochemical-derived products. One such chemical, 3-hydroxypropionic acid (3-HP), is an organic acid that can be chemically converted to acrylic acid, 1,3-propanediol, and malonic acid, among others (della Pina et al., 2011), offering a great potential for bioproduction of a wide range of polymers. In fact, 3-HP has been included in the list of the top platform chemicals that can be obtained from biomass (Werpy and Petersen, 2004).

3-HP can be naturally produced by several bacteria through different biosynthetic routes. Among them, the glycerol-dependant pathway, and the pathways *via* malonyl-CoA and β-alanine intermediates have been the most extensively investigated (Kumar et al., 2013; de Fouchécour et al., 2018). Multiple microorganisms have been engineered to produce 3-HP, including bacteria (Jers et al., 2019) and yeast (Ji et al., 2018). The highest 3-HP concentrations and productivities achieved so far have been obtained in *Escherichia coli* [76.2 g L^−1^ of 3-HP and 1.89 g L^−1^ h^−1^, respectively (Kim et al., 2020)] and *Klebsiella pneumoniae* (Zhao et al., 2019). Both bacterial species were engineered to produce 3-HP through the glycerol-dependent pathway. This facilitated the use of glycerol as a carbon source and, ultimately, of crude glycerol, which is an abundant, inexpensive and renewable feedstock produced as a main by-product of the conventional biodiesel production process. Morevover, glycerol is very attractive for production of organic acids such as 3-HP due to its higher degree of reduction compared to glucose.

Production of 3-HP in yeast has been mainly pursued by introducing the malonyl-CoA or β-alanine pathways, e.g., reaching a titer of about 13.7 g L^−1^ and with a 0.14 ± 0.0 C-mol^−1^ yield on glucose in *Saccharomyces cerevisiae* fed-batch cultures (Borodina et al., 2015). The methylotrophic yeast *Pichia pastoris* (syn*. Komagataella phaffii*) is able to grow efficiently on glycerol. Furthermore, crude glycerol may contain from 1% to 25% w/w methanol (Luo et al., 2016), making this feedstock ideal for a methylotrophic microorganism such as *P. pastoris*. In addition, this yeast can grow at a pH as low as 3 (Cregg et al., 1993; Werten et al., 1999), which allows performing the fermentation process at a pH below the pKa of 3-HP (4.51). This would enable the use of *in situ* product recovery systems, thereby avoiding 3-HP accumulation to toxic levels and facilitating 3-HP export from cells (van Maris et al., 2004). The 3-HP extraction yield using a membrane-assisted *in situ* product recovery system was increased from 5% to 74% when the pH of the medium was decreased from 5 to 3.2 (Chemarin et al., 2019).

**FIGURE 1:**
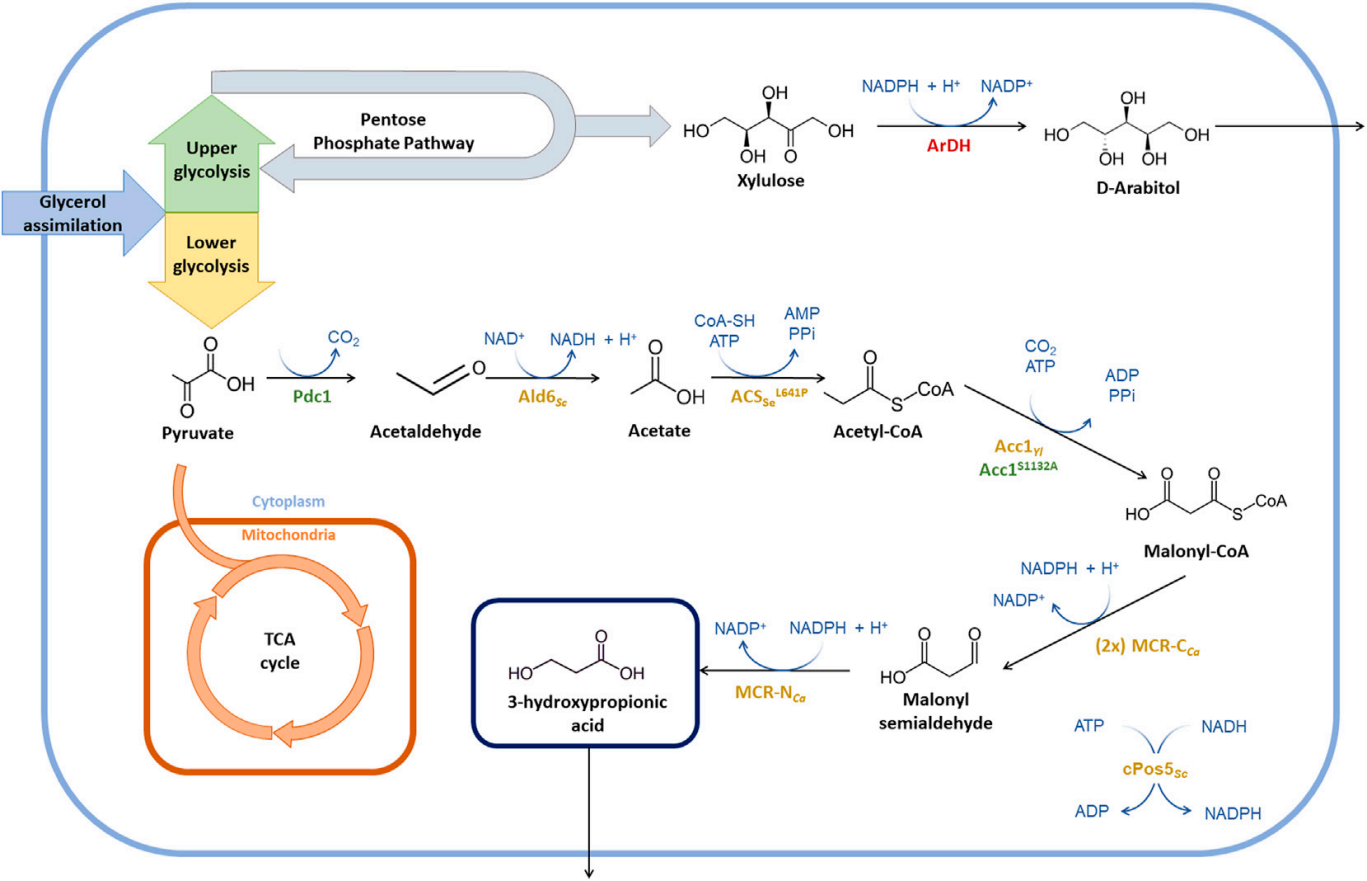
Summary of metabolic engineering strategies for the optimisation of 3-HP production in *P. pastoris*. Strain engineering started from the 3-HP producing strain PpHP6 containing the malonyl-CoA to 3-HP pathway (Fina et al., 2021). Heterologous enzymes are depicted in yellow, endogenous enzymes being overexpressed are shown in green, and the enzymes catalyzing the reactions of genes being deleted are shown in red. The (2x) indicates that a second copy of the MCR-C_Ca_ gene was introduced. Enzyme abbreviations: Pdc1, pyruvate decarboxylase; Ald6_*Sc*_, aldehyde dehydrogenase from *S. cerevisiae*; ACS_*Se*_^L641P^, acetyl-CoA synthase from *S. enterica* with the mutation L641P; Acc1_*Yl*_, acetyl-CoA carboxylase from *Y. lipolytica*; Acc1^S1132A^, acetyl-CoA carboxylase from *P. pastoris* with the mutation S1132A; MCR-C_*Ca*_, C-terminal domain of the malonyl-CoA reductase from *C. aurantiacus*; MCR-N_*Ca*_, N-terminal domain of the malonyl-CoA reductase from *C. aurantiacus*; cPos5_*Sc*_, NADH kinase from *S. cerevisiae* relocated into the cytoplasm; ArDH, D-arabitol dehydrogenase.

In a recent study, we addressed the use of *P. pastoris* to produce 3-HP from glycerol (Fina et al., 2021). This yeast was metabolically engineered to express the malonyl-CoA reductase pathway, which consists of two consecutive steps reducing malonyl-CoA into 3-HP, while consuming two NADPH molecules (Figure 1). Specifically, we expressed the gene encoding for the bi-functional enzyme malonyl-CoA reductase (MCR) from *Chloroflexus aurantiacus.* This pathway provides the simplest way to produce 3-HP from glycerol in *P. pastoris*, as the glycerol-dependant pathway is coenzyme B12-dependant. Since *P. pastoris* does not naturally produce coenzyme B-12, the introduction of the glycerol-dependant pathway for 3-HP biosynthesis pathway in this cell factory would require either the addition of such expensive cofactor to the fermentation medium or the co-expression of the multistep B-12 biosynthetic pathway (Fang et al., 2018).

In addition, several improvements were made to the original strain. First, the independent expression of the two MCR subunits was implemented, leading to a 12.5-fold increase in 3-HP production. Second, the resulting strain was further engineered to increase the availability of NADPH and malonyl-CoA, which are the two substrates of this route. To this end, the acetyl-CoA carboxylase gene from *Yarrowia lipolytica* (*ACC1*_*Yl*_) and a NADH kinase from *Saccharomyces cerevisiae* (*cPOS5*_*Sc*_) were expressed in the cytosol. However, this strategy resulted in rather modest improvements in terms of product yield, pointing to the delivery of malonyl-CoA as a major bottleneck to further improve 3-HP production. Notably, this strain produced up to 24.75 g L^−1^ of 3-HP in 45.5 h in a fed-batch culture using glycerol as a carbon source, which is, to our knowledge, the highest productivity reported so far in yeast (0.54 g L^−1^ h^−1^) (Fina et al., 2021).

Despite providing the first demonstration of the potential of *P. pastoris* for 3-HP production from glycerol, 3-HP production metrics from our previous study are still far from being industrially-relevant, as a minimum productivity of 2.5 g L^−1^ h^−1^, 50∼100 g L^−1^ and >0.5 g/g yield are generally required to achieve an economically feasible bioprocess for carboxylic acid production (Werpy and Petersen, 2004; Mazzoli, 2021).

Here, further metabolic engineering of the previously developed 3-HP-producing strains (Fina et al., 2021) was performed to increase the production metrics. In particular, we aimed at improving both the biosynthetic pathway for 3-HP from its metabolic precursor (acetyl-CoA), as well as improving the metabolic flux towards acetyl-CoA synthesis and reducing by-product (arabitol) formation. Notably, we used three different small-scale cultivation systems for better characterisation of the impact of genetic modifications on cell growth and product yields and subsequent transfer to bioreactor-scale cultivations.

## Materials and Methods

### Molecular Biology and Strains

All the strains used in this study are listed in Table 1. A detailed description of the molecular biology workflow to generate each strain can be found in the Supplementary Material S1. The modular cloning vectors GoldenPics [(Prielhofer et al., 2017); Addgene Kit #1000000133] and the vectors for CRISPR-Cas9 of the CrisPi kit (Gassler et al., 2019); Addgene Kit #1000000136) were used.

**TABLE 1:** Strain list.

Strain name	Parental strain	Additional expression cassettes or deleted genes compared to parental strain	Source
X-33			Invitrogen-Thermo Fisher Scientific (MA, United States)
PpHP2	X-33	pGAP_*mcr-N*_*Ca*_ *_*AOX1tt pGAP_*mcr-C*_*Ca*_ *_*AOX1tt	Fina et al. (2021)
PpHP6	PpHP2	pGAP___ *ACC1*_*Y*l__ScCYC1tt pGAP_*cPOS5*_*Sc_*_RPS3tt	Fina et al. (2021)
PpHP7	PpHP2	pGAP_*mcr-C*_*Ca*_ *_*TDH3tt	This study
PpHP9	PpHP7	pGAP_ACC1^*S1132A*^_AOX1tt	This study Plasmid source (Liu et al., 2019); Addgene #126740
PpHP11	PpHP7	pTEF1_*acs*_*Se*_^*L641P*^_RPS3tt	This study
PpHP12	PpHP7	pTEF1_*acs*_*Se*_^*L641P*^_RPS3tt pMDH3_*ALD6*_*Sc*__TDH3tt	This study
PpHP8	PpHP6	pGAPeN_ *mcr-C*_*Ca*_ *_*TDH3tt	This study
PpHP13	PpHP8	pTEF1_*acs*_*Se*_^*L641P*^_RPS3tt	This study
PpHP14	PpHP8	pTEF1_*acs*_*Se*_^*L641P*^_RPS3tt pMDH3_*ALD6*_*Sc*__TDH3tt	This study
PpHP15	PpHP8	∆*ArDH*	This study
PpHP16	PpHP8	∆*ArDH* pPDC1_*PDC1_*PDC1tt	This study
PpHP17	PpHP13	∆*ArDH*	This study
PpHP18	PpHP13	∆*ArDH* pPDC1_*PDC1_*PDC1tt	This study

The strains generated using the GoldenPics plasmids were generated by integration of the heterologous DNA at the loci described in the Supplementary Material S1.

For the new strains generated by CRISPR-Cas9, the integration cassette was amplified from *P. pastoris* genome using a high-fidelity Q5 polymerase (New England Biolabs, MA, United States) and the recommended protocol (Gassler et al., 2019). The integration loci are also indicated in the Supplementary Material S1. The integrity of the inserted cassette was verified by Sanger sequencing at the Genomics and Bioinformatics Service of the Universitat Autònoma de Barcelona.

### Copy Number Determination by Droplet Digital PCR

The number of copies of the gene *mcr-C*_*Ca*_ of the highest producing clones of the strains PpHP7 and PpHP8 was tested with droplet digital PCR (ddPCR) following a previously reported method (Cámara et al., 2016), except that the QX200^TM^ ddPCR^TM^ EvaGreen Supermix (Biorad, CA, United States) was used instead of Taqman probes. First, the genomic DNA of the highest producing clones was purified using the Wizard® Genomic DNA Purification Kit from Promega (WI, United States). Afterwards, 0.5 µg of genomic DNA were restricted using the restriction enzymes BamHI-FD and EcoRI-FD from Thermo Fisher Scientific (MA, United States). Subsequently, the genomic DNA was diluted to a concentration of 1 ng μL^−1^.

Second, a master mix of 22.5 µL was prepared with the forward primer at 0.4 µM, the reverse primer at 0.2 µM, and the restricted genomic DNA at 0.08 ng μL^−1^. Afterwards, the master mix was mixed with 22.5 µL of EvaGreen 2X master solution and was thoroughly mixed by vortexing. Following droplet generation using a Droplet generator (Biorad, CA, United States), the droplets were transferred to a 96-tubes rack and subsequently sealed with a PCR Plate Heat Seal. The PCR was carried using a C1000 Touch Thermal Cycler (Biorad, CA, United States). The annealing temperature was set to 60°C. Finally, the ddPCR results were analysed using a QX200 Droplet Digital PCR system (Biorad, CA, United States).

The primers used for ddPCR are listed in Table 2. The primer pairs ddPCR_mcrCCa_FW and ddPCR_mcrCCa_RV (Table 2) were used to amplify the *mcr-C*_*Ca*_ gene. Primers ddPCR_act_FW and ddPCR_act_RV were used to amplify the β-actin (*ACT1)* endogenous gene, which was used as reference of a single-copy gene.

**TABLE 2:** Oligonucleotides used for gene copy number determination analysis with ddPCR.

Primer name	Primer sequence
ddPCR_mcrCCa_FW	CCT​AAC​GAT​GTT​GCT​GCT​TTG​GAG
ddPCR_mcrCCa_RV	GGA​TCA​GGT​GGA​TTA​GGC​AAG​TTA​GC
ddPCR_act_FW	TCC​GGT​GGT​ACT​ACT​ATG​TTC​C
ddPCR_act_RV	GAT​AGA​ACC​ACC​GAT​CCA​TAC​G

### 24-Deep-Well Plates Screening

*P. pastoris* strains were inoculated into 50 mL falcon tubes containing 5 mL of YPG (1% yeast extract, 2% peptone and 1% v/v glycerol) supplemented with 100 μg mL^−1^ zeocin (InvivoGen, CA, United States). The cells were grown overnight at 30°C and 200 rpm in an incubator shaker Multitron Standard (Infors HT, Bottmingen, Switzerland) with a 2.5 cm orbit. 50 μL of overnight-grown cultures were used to inoculate each well of a 24 deep-well plate containing 2 mL of Buffered Minimal Glycerol (BMG) medium, containing 100 mM potassium phosphate buffer pH 6, 1.34% yeast nitrogen base (YNB), 1% v/v glycerol, and 0.4 mg L^−1^ biotin. The cultures were incubated at 28°C and 220 rpm in the same incubator shaker using a platform with a slope of 20° to improve the aeration. The cultures were grown for 48 h to ensure the full consumption of the substrate. Thereafter, culture samples were centrifuged at 12,000 g for 5 min and filtered through a 0.20 µm pore size syringe filter (SLLGX13NK, Merck Millipore, CA, United States). The 3-HP was quantified using HPLC.

The parental clones PpHP2 and PpHP6 were screened in triplicates. Six to eight clones were screened in triplicates for the strains generated by single-homology integration (PpHP7, PpHP8, and PpHP9). One single clone was screened in triplicates for the strains generated using CRISPR-Cas9.

### Small-Scale Screening in Falcon Tubes Using FeedBeads®

The inoculum was prepared following the same protocol described for the deep-well plates screenings. Afterwards, 50-mL falcon tubes were filled with 5 mL of Buffer Minimal medium (BM; 100 mM potassium phosphate buffer pH 6, 1.34% YNB and 0.4 mg L^−1^ biotin), supplemented with one Glycerol FeedBeads® (SMFB12001, Kuhner Shaker GmbH, Germany). This FeedBead® releases 40 mg of glycerol in 48 h. The cultures were inoculated with 50 µL of the overnight saturated cultures. The falcon tubes were incubated in an incubator shaker at 200 rpm and 30°C for 48 h. Each clone was tested in triplicate. A triplicate control was performed by adding one FeedBead® to a falcon with 5 mL of BM medium. These controls were used to determine the actual release of glycerol under the tested conditions.

Culture samples were centrifuged at 12,000 g for 5 min and filtered through a 0.20 µm pore size syringe filter (SLLGX13NK, Merck Millipore). The 3-HP was quantified using HPLC as described below.

### Mini Bioreactors Screening

The automated cultivation and sampling platform described elsewhere was used (Heux et al., 2014).

The bioreactor medium contained 2.5 g L^−1^ glycerol, 1.8 g L^−1^ citric acid, 0.02 g L^−1^ CaCl_2_ · 2 H_2_O, 12.6 g L^−1^ (NH_4_)_2_HPO_4_, 0.5 g L^−1^ MgSO_4_ · 7 H_2_O, 0.9 g L^−1^ KCl, 50 μL antifoam Glanapon 2000 kz (Bussetti and Co., GmbH, Vienna, Austria), 0.4 mg L^−1^ biotin and 4.6 mL L^−1^ of PTM1 trace salts (Maurer et al., 2006). The pH was set to 5 using 1 M HCl. The medium was autoclaved without the trace salts and the biotin, which were filter sterilized and added to the medium under sterile conditions. Each mini bioreactor was filled with 15 mL of medium. The pre-inoculum was prepared as described for the other two screening methods (deep-well plates and falcon tubes with FeedBeads®). The overnight-saturated cultures were used to inoculate 250 mL shake flasks with 25 mL of YPG at a starting OD_600_ of 0.5–1.5. The cells were grown for 8 h at 28°C and 160 rpm in a shaker incubator to ensure the cells were at the exponential phase. Each bioreactor was inoculated at a starting OD_600_ of 0.025. Each strain was tested in triplicate.

The temperature of the bioreactors was set to 28°C. The dissolved oxygen (DO) and the pH were monitored throughout the cell culture. Samples were automatically withdrawn from the bioreactors every 2 h for the first 16 h of culture to monitor the OD_600_. After 16 h, samples were withdrawn hourly during 8 h for both OD_600_ monitoring and supernatant analysis. The 250 µL samples for culture supernatant analysis were automatically placed on 96-well plates with a 0.45 µm pore size filter bottom. The samples were filtered by applying vacuum. Glycerol, 3-HP, and D-arabitol were quantified using NMR as described below.

The growth rate (µ_max_), the specific substrate consumption rate (qS_Glyc_), and the specific 3-HP and D-arabitol production rates (qP_3HP_ and qP_Abt_) were calculated during the exponential growth phase using the R program PhysioFit (Peiro et al., 2019).

### Fed-Batch Cultures in Bioreactors

The cultures were carried out using a DASGIP Parallel Bioreactor System (Eppendorf, Germany). The starting volume of each 1.3 L reactor vessel was 400 mL. The Batch Medium described for the mini bioreactors with 40 g L^−1^ of glycerol was used. All the medium components except the biotin and the trace salts were mixed, placed into the reactor, and autoclaved. Biotin and trace salts were added through the septum port after autoclaving the reactor. The pH was controlled at 5 using 15% ammonia (only base addition was used). Aeration was set to 1 vvm (0.4 L min^−1^). The DO was set to 30%, and it was automatically controlled using the following cascade: 1) Increasing the stirring rate from 400 to 1,200 rpm; 2) Compressed air was mixed with pure oxygen to increase the percentage of oxygen of the inlet gas. The reactors were inoculated at a starting OD_600_ of 1. The inocula were prepared as described elsewhere (Garcia-Ortega et al., 2013).

The feeding medium composition was 400 g L^−1^ glycerol, 10 g L^−1^ KCl, 6.45 g L^−1^ MgSO_4_ · 7 H_2_O, 0.35 g L^−1^ CaCl_2_ · 2 H_2_O, 0.2 mL L^−1^ antifoam Glanapon 2,000 kz, 1.2 mg L^−1^ biotin and 15 mL L^−1^ PTM1 trace salts. All the feeding medium components except the biotin and the trace salts were mixed and autoclaved. Biotin and trace salts were filter-sterilized and added to the feeding medium under sterile conditions. Feeding medium was added to the bioreactor using the exponentially increasing feed-rate described by Eq. 1, where F(t) is the feeding rate at every time, t_0_ is the time where the feeding starts (end of the batch phase), X (t_0_) is the biomass concentration at the end of the batch phase, V (t_0_) is the starting volume of the reactor (the volume for the batch phase), $Y_{X/S}$ is the biomass to substrate yield, and S_0_ is the concentration of substrate in the feeding medium. Eq. 1 was simplified by assuming $Y_{X/S}$ would not vary significantly between the batch and the fed-batch phase (Eq. 2). In Eq. 2, S_batch_ is the concentration of substrate for the batch medium (40 g L^−1^).

$$F\left(t\right)=\;\frac{µ\left[X\left(t_{0}\right)V\left(t_{0}\right)\right]}{Y_{X/S}S_{0}}e^{\left[µ\left(t-t_{0}\right)\right]}$$ (1)

$$X\left(t_{0}\right)=Y_{X/S}S_{batch}\;$$ (2)

Therefore, the flow rate of the feeding medium was set using Eq. 3.

$$F\left(t\right)=\;\frac{µS_{batch}V\left(t_{0}\right)}{S_{0}}e^{\left[µ\left(t-t_{0}\right)\right]}$$ (3)

The feeding started once the DO increased above 60%, indicating that all the substrate of the batch phase had been consumed. Samples were collected for biomass cell dry weight (CDW) determination and supernatant analysis. The CDW was quantified in triplicates. From 0.5 to 2 mL of culture were filtered through pre-weighted glass microfiber filters (APFF04700, Merck Millipore). The filters were then washed with 10 mL of distilled water with 9 g L^−1^ NaCl and dried overnight at 105°C. The filters containing the dry biomass were weighted to calculate the CDW.

Culture samples were centrifuged 5 min at 12,000 g and the supernatant was filtered with a 0.2 µm pore size syringe filter (SLLGX13NK, Merck Millipore). The filtered supernatant was analysed with HPLC for glycerol, 3-HP, and D-arabitol quantification.

### HPLC Analysis

HPLC was used to quantify the glycerol, the D-arabitol, and the 3-HP from the supernatant of the deep-well plates, the FeedBeads® screening, and the fed-batch bioreactor samples. A previously described HPLC protocol was used (Fina et al., 2021). The 3-HP was quantified from the UV spectrum. Glycerol and D-arabitol were quantified from the Refractive Index (RI) spectrum. As 3-HP and glycerol have the same retention time and 3-HP is also detected on the RI detector, the area corresponding to the 3-HP (previously quantified from the UV signal) was subtracted to latter calculate the actual glycerol concentration.

### NMR Analysis

Glycerol, D-arabitol, and 3-HP were quantified from the culture supernatants of the mini bioreactor cultures using 1D-1H Nuclear Magnetic Resonance (NMR) on a Bruker Advance III 800 MHz spectrometer (Bruker BioSpin, Germany). Prior to the analyses, 180 µL of filtered culture supernatant samples were mixed with 20 µL of 10 mM TSP (3-(trimethylsilyl)-[2,2,3,3-^2^H_4_]-propionic acid sodium salt), which was used as an internal standard. The NMR spectrometer was coupled to a 5 mm CQPI cryoprobe, which was set to 280 K. A 30° presaturation pulse was recorded, followed by a relaxation delay of 7 s. The software TopSpin 3.6.4 (Bruker BioSpin, Germany) was used for the integration of the peaks.

## Results and Discussion

### Increasing *mcr*-*C*_*Ca*_ Copy Number Leads to Higher 3-HP Production

Previous results showed that the co-overexpression of *ACC1* and *cPOS5* in the initial 3HP-producing strain PpHP2 (Table 1) led to a small but significant increase in the 3-HP yield on glycerol in strain PpHP6 (from 0.131 Cmol Cmol^−1^ to 0.146 Cmol Cmol^−1^) (Fina et al., 2021). However, the overexpression of either *ACC* or *cPOS5* did not result into any further significant impact on 3-HP production, highlighting the malonyl-CoA reductase step as a major bottleneck for 3-HP production, rather than the supply of malonyl-CoA and NADPH availability.

**FIGURE 2:**
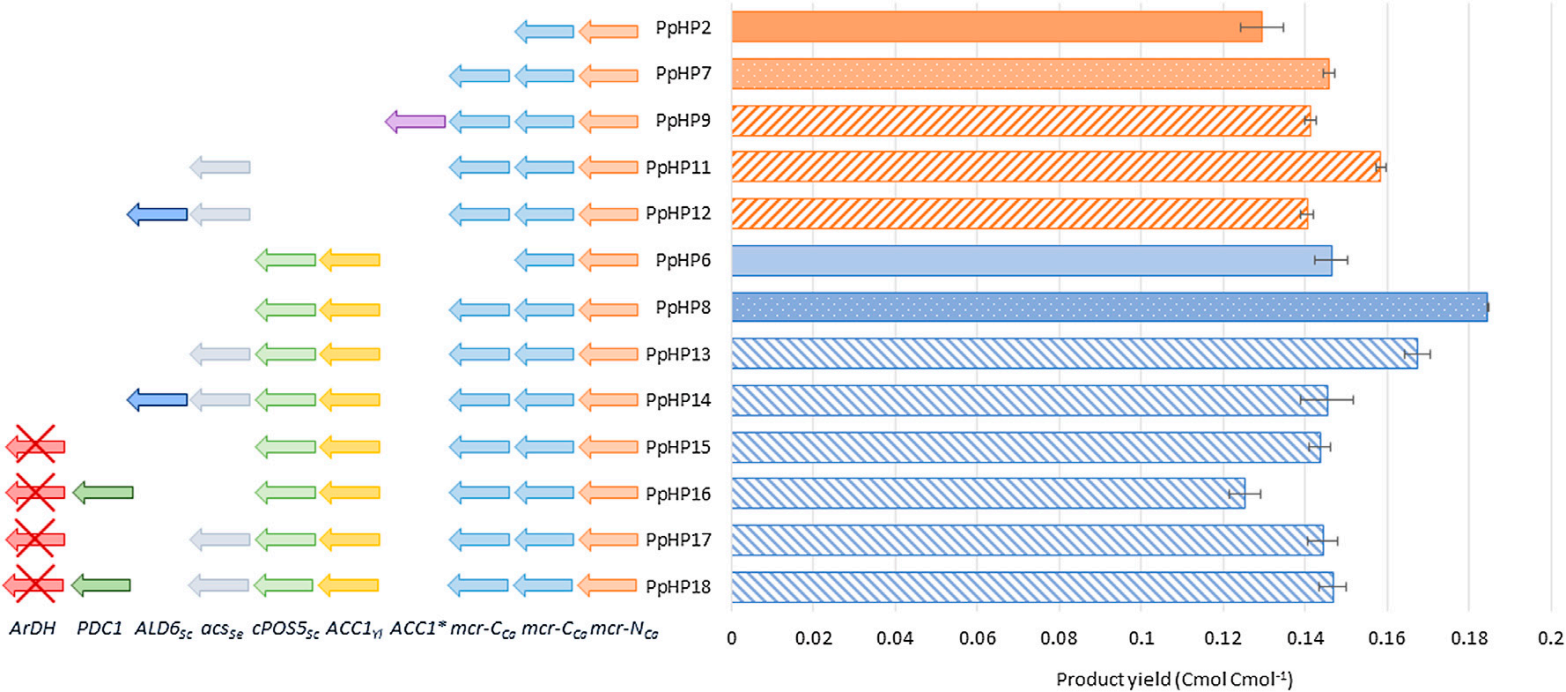
3-HP production yield of the recombinant *P. pastoris* strains tested in deep-well plates. The genetic modifications performed to each strain are shown at the left side of the graph. The product yield in Cmol^−1^ is shown at the right side of the graph.

Thereby, a second copy of the C-terminal domain of the malonyl-CoA reductase gene (*mcr-C*_*Ca*_) under the control of the strong and constitutive GAP promoter was added to the PpHP2 and PpHP6 strains, generating strains PpHP7 and PpHP8, respectively. For each strain, six independently isolated clones were tested in triplicate using deep-well plates and BMG medium. The highest producer clone for strains PpHP7 and PpHP8 produced 1.81 ± 0.02 g L^−1^ and 2.29 ± 0.01 g L^−1^ of 3-HP (i.e., 0.143 ± 0.001 and 0.180 ± 0.001 Cmol^−1^ yield on glycerol, respectively). The gene copy analysis showed that both clones contained 2 copies of *mcr-C*_*Ca*_. The 3-HP yield achieved by the strains PpHP2 and PpHP6 was 0.130 ± 0.005 and 0.146 ± 0.004 Cmol^−1^, respectively. Therefore, the addition of a second copy of *mcr-C*_*Ca*_ to PpHP2 and PpHP6 led to a 1.38 and 1.23-fold improvement, respectively (*p-values* of 0.01 and 0.0001), as shown in Figure 2.

These results are consistent with previous studies with other yeasts such as *S. cerevisiae* or *Schizosaccharomyces pombe*. The use of a multicopy integrative vector containing the *mcr*_Ca_ and the *ACC1* gene from *S. cerevisiae* led to a 3-fold increase in 3-HP production in *S. cerevisiae* (Kildegaard et al., 2016). In *S. pombe,* the addition of a second copy of *mcr-C*_*Ca*_ led to an almost 3-fold improvement in the 3-HP yield. Addition of a third copy of *mcr-C*_*Ca*_ led to a further 2-fold improvement in 3-HP production, while the addition of a fourth copy of the same gene, or the addition of a second copy *mcr-N*_*Ca*_ did not have an impact on the final 3-HP titre (Takayama et al., 2018).

Nevertheless, the increase in 3-HP production achieved in *P. pastoris* when additional copies of the *mcr-C*_*Ca*_ gene were introduced was significantly lower than the one achieved in other yeasts. Notably, the MCR specific activity of cell extracts of *P. pastoris* expressing *mcr*_*Ca*_ under the control of pGAP was more than one order of magnitude higher than the same gene expressed under the control of pTEF1 in *S. cerevisiae* (Chen et al., 2014; Fina et al., 2021). Additional bottleneck(s) upstream of the malonyl-CoA reductase reaction steps are probably limiting the 3-HP production, so no further copies of *mcr-C*_*Ca*_ were introduced.

### Expression of the de-regulated *ACC1* ^S1132A^ Mutant Does Not Improve 3-HP Production

The acetyl-CoA carboxylase (Acc) catalyses the conversion of acetyl-CoA into malonyl-CoA. The enzyme Acc1^S1132A^ from *P. pastoris* contains a site mutation (S1132A) that prevents the post-translational inactivation of the enzyme by phosphorylation when glucose is depleted (Choi and da Silva, 2014). Expression of this endogenous *ACC1* variant under the control of the GAP promoter showed a positive impact on the production of acetyl-CoA derived drugs in *P. pastoris* (Liu et al., 2019). The same approach was used in PpHP7, but the new strain (PpHP9) produced less 3-HP than the parental strain (0.138 ± 0.001 Cmol^−1^, Figure 2).

Acc1 is organized as a homodimer. The phosphorylation of one of the two monomers inactivates the enzymatic complex (Hunkeler et al., 2016). Therefore, it is plausible that the co-existence of the heterologous mutated version of Acc1 and the endogenous non-mutated versions leads to the formation of inactive heterodimers, hindering the effect of Acc1^S1132A^ overexpression. This hypothesis is supported by previous studies showing that replacement of the endogenous *ACC1* sequence with *ACC1* ^S1132A^ was required to ensure that Acc1 actvity would only be controlled by Acc1^S1132A^ expression levels (Wen et al., 2020). Deletion of the endogenous *ACC1* gene in strains PpHP9 may thus had a positive effect on 3-HP production. Moreover, it is well reported that Acc1 is a highly regulated enzyme (Hunkeler et al., 2016; Chen et al., 2018). In addition to its post-translational regulation, this enzyme is also regulated by allosteric inhibition (Goodridge, 1972; Qiao et al., 2015). Differences in the kinetic parameters between the two Acc1 variants might also explain the differences in the final 3-HP yield.

In contrast, the overexpression of *ACC1* from *Y. lipolytica* in *P. pastoris* led to a small increase in 3-HP production (Fina et al., 2021). The yield in PpHP8 (harboring Acc1_*Yl*_) was around 1.25-fold higher than in PpHP7. These results are consistent with recent studies where the heterologous expression of *ACC1* from *Y. lipolytica* yielded a higher accumulation of malonyl-CoA than the overexpression of the endogenous *S. cerevisiae ACC1* (Pereira et al., 2022).

### Metabolic Engineering for Increased Acetyl-CoA Supply and Minimization of By-Product Formation

The endogenous cytosolic acetyl-CoA biosynthesis pathway was overexpressed to pull the conversion of pyruvate into acetyl-CoA. To this end, a modified Acetyl-CoA synthase from *Salmonella enterica* was used (*acs*_*Se*_^*L641P*^). The gene harbours a mutation (L641P) to suppress the post-translational inhibition of the enzyme by acetylation (Shiba et al., 2007). Expression of *acs*_*Se*_^*L641P*^ was tested with or without the co-expression of the aldehyde dehydrogenase gene *ALD6* from *S. cerevisiae*. The two genes were expressed under the control of the TEF1 and MDH3 promoters, respectively, which are two mid-to-high expression constitutive promoters (Prielhofer et al., 2017). Use of pGAP was dismissed to avoid the dilution of the expression of the other genes, as there are already 5 heterologous genes under the control of pGAP (Marx et al., 2009; Aw and Polizzi, 2013).

Expression of *acs*_*Se*_ in strains PpHP7 and PpHP8, generating strains PpHP11 and PpHP13, led to opposite effects. While PpHP11 produced significantly more 3-HP (0.153 ± 0.002 Cmol^−1^ yield) than PpHP7 (*p-value* of 0.0003), its expression in PpHP13 led to a reduction of the yield (*p-value* 0.0006), as shown in Figure 2.

The expression of the aldehyde dehydrogenase *ALD6* in both PpHP11 and PpHP13 (PpHP12 and PpHP14, respectively) led to a decrease in the 3-HP yield for both strains (Figure 2). Interestingly, the deletion of *ALD6* in *S. cerevisiae* led to a reduced susceptibility to 3-HP, as Ald6 might catalyse the conversion of 3-HP into the toxic compound 3-hydroxypropanaldehyde (3-HPA) (Kildegaard et al., 2014). Thus, it is plausible that *ALD6* overexpression in 3-HP producing *P. pastoris* strains leads to an increased accumulation of 3-HPA.

Finally, the overexpression of endogenous pyruvate decarboxylase (*PDC1*) was tested. To this end, an additional copy of *PDC1* under the control of its endogenous promoter was added to the genomes of the strains PpHP8 and PpHP13. Addition of a second copy of *PDC1* was coupled to the deletion of the *ArDH* gene, which is reported as the main responsible of the D-arabitol production in *P. pastoris* (Melo et al., 2020). To distinguish the effect of the deletion of *ArDH* from the overexpression of *PDC1*, strains PpHP15 and PpHP17 were generated by deleting *ArDH* from strains PpHP8 and PpHP13, respectively (without an additional copy of *PDC1*). The strains PpHP16 and PpHP18 were also obtained from PpHP8 and PpHP13, and they harbour both the deletion of *ArDH* and a second copy of the endogenous *PDC1*. None of the new strains produced more 3-HP than their parental strains (Figure 2).

### Screening Under Substrate-Limiting Conditions Yields Different Ranking of Strains Compared to Conventional Substrate-Excess Screening Strategies

Previous results obtained in deep-well plates showed that the overexpression of the cytosolic acetyl-CoA production pathway did not result in a significant increase in the final 3-HP production. Moreover, accumulation of D-arabitol was not observed in none of the strains, as was the case for PpHP6 strain in fed-batch cultivations (Fina et al., 2021). Thus, the effect of *ArDH* deletion could not be properly assessed at small scale. Therefore, a representative subset of the strains constructed in this study were further cultured in two additional systems: 1) Mini bioreactors, favouring fully aerobic conditions and easy withdrawal of multiple samples. 2) Falcon tubes containing FeedBeads®, which release glycerol at a low and constant rate (40 mg in 48 h), thereby permitting cell growth at a low rate, i.e., under substrate-limiting conditions resembling a fed-batch process.

The mini bioreactors were sampled during the exponential phase, which allowed to accurately determine the µ_max_ and the q-rates of the consumption of glycerol and the production of 3-HP and D-arabitol of the different strains, whereas endpoint samples (at 48 h) were taken from cultivations in falcon tubes containing FeedBeads®.

**FIGURE 3:**
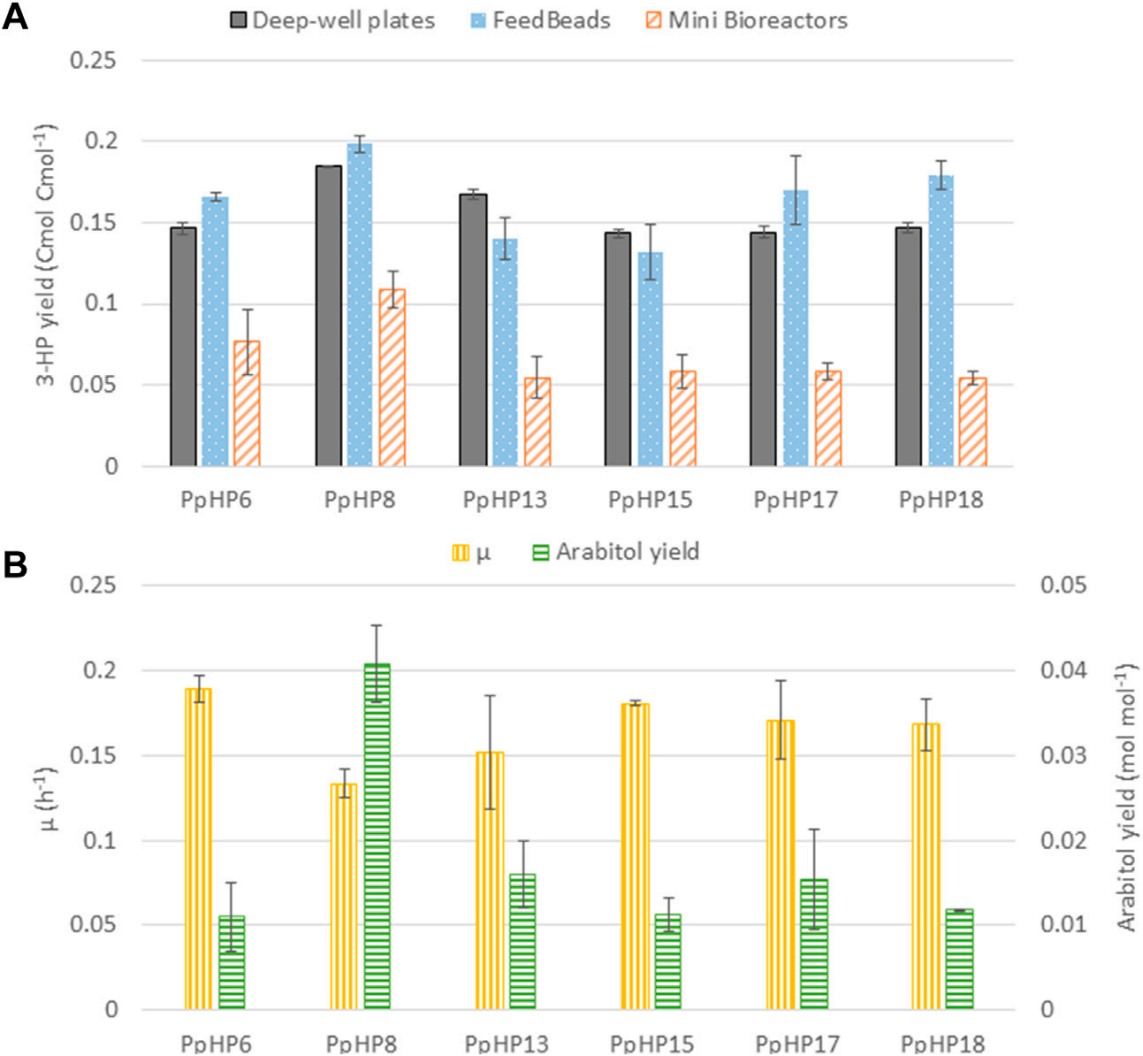
Comparison of the most relevant 3-HP producing *P. pastoris* strains under three different screening conditions. **(A)** 3-HP production yield in deep-well plates (grey solid bars), FeedBeads® (blue dotted bars), and mini bioreactors (orange striped bars). **(B)** Growth rate (yellow vertically striped bars) and arabitol production yield (green horizontally striped bars) for each strain grown in mini bioreactors.

Notably, comparison of the results from these alternative screening conditions with the deep-well plate cultivations (Figure 3) reveals that the ranking of the strains based on their product yield varies depending on the screening condition. PpHP8 exhibited the highest product yield for the three screening conditions. However, the combined effect of ACS and Pdc1 overexpression and *ArDH* deletion (strain PpHP18) was clearly growth condition dependent. Indeed, the 3-HP production of the two strains, PpHP8 and PpHP18 was very similar at a low growth rate (i.e., using FeedBeads®), but differed significantly at µ_max_ (i.e., in the mini bioreactor experiment). Interestingly, 3-HP yields were generally higher in deep-well plates and falcon tubes, where oxygen transfer is usually considered as rate-limiting during cultivation, than in fully aerobic mini bioreactor cultures. This is consistent with the fact that the pGAP promoter is upregulated in *P. pastoris* cells growing on glucose under hypoxic conditions (Baumann et al., 2011).

Moreover, strains PpHP13, PpHP15, PpHP17, and PpHP18 showed a much lower 3-HP product yield than PpHP8 when the cells were grown in mini bioreactors. Conversely, the µ_max_ of strain PpHP8 was the lowest of this subset of stains. An inverse correlation between µ_max_ and 3-HP product yield was observed. Despite lower growth rates would be generally expected for the strains with a larger number of heterologously overexpressed genes, overexpression of ACS and Pdc1 and the deletion of *ArDH* led to a higher µ_max_. Acc1 overexpression has been reported to cause growth impairment in yeast (Shi et al., 2014; Kildegaard et al., 2016). This reduction in the growth rate was attributed to a metabolic burden. The fact the cell growth improves when the cytosolic acetyl-CoA pathway is overexpressed suggests that the lower growth rate of PpHP8 might be caused by a limitation in acetyl-CoA supply for the biosynthetic reactions. Such hypothesis is supported by the fact that the product yield of strains PpHP8 and PpHP18 are almost identical when the cells are grown at a low growth rate (i.e., using FeedBeads®). This is also reinforced by the differential D-arabitol production of strains PpHP8 and PpHP13. The presence of arabitol denotes a redox imbalance (Fina et al., 2021). When the delivery of acetyl-CoA is improved by overexpressing ACS (PpHP13), the D-arabitol production decreases.

Notably, the mini bioreactors data reveals that the deletion of *ArDH* from strain PpHP8 (strain PpHP15) resulted in a decrease in the D-arabitol yield and an improvement of the µ_max_. Production of D-arabitol from glycerol results in net ATP consumption. Thus, the reduction in the D-arabitol production leads to higher energetic yield from the substrate, as well as a better carbon conservation (as less by-product D-arabitol is produced), thereby potentially increasing acetyl-CoA supply and supporting higher growth rates. However, this might result in reduced Acetyl-CoA availability for 3-HP production.

Altogether, the observed results indicate the supply of cytosolic acetyl-CoA as a major limiting factor to further increase the 3-HP yield.

### Fed-Batch Cultures

**FIGURE 4:**
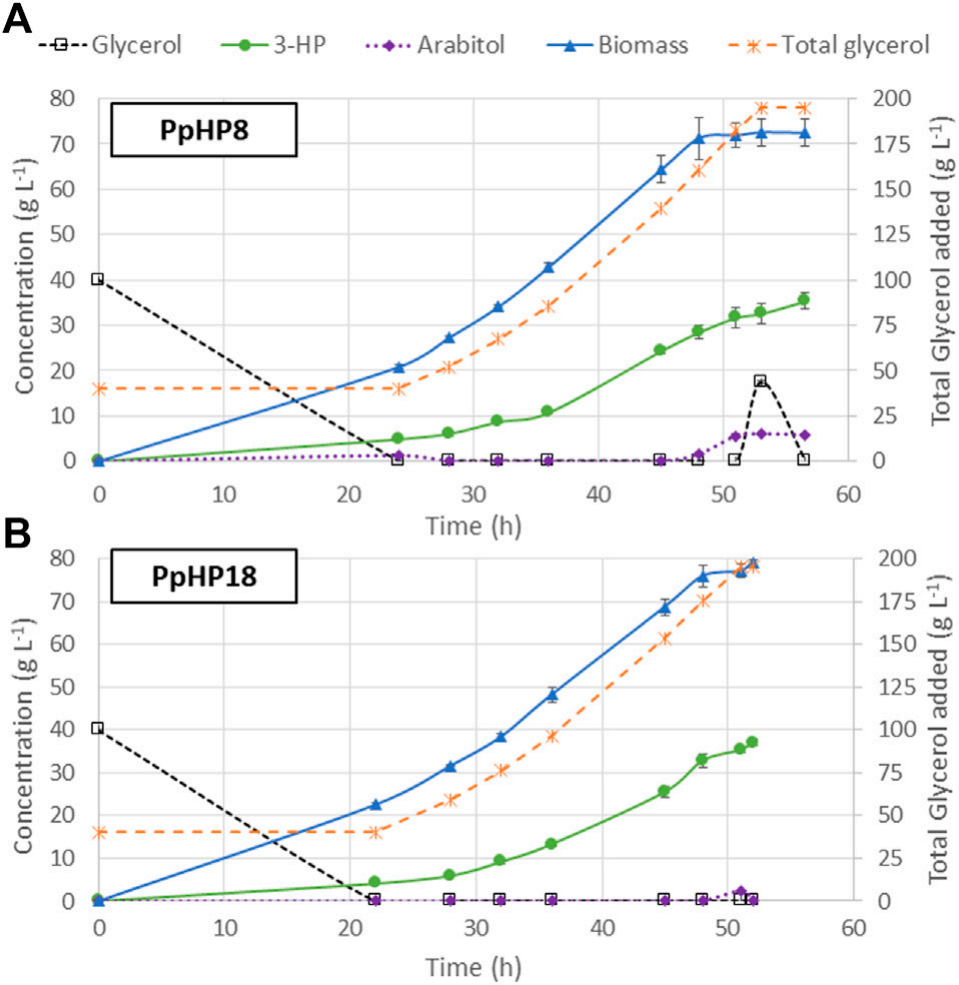
Profiles of fed-batch cultivations of the PpHP8 **(A)** and PpHP18 **(B)** strains. Concentration of glycerol, biomass, 3-HP, and D-arabitol are represented using the left-side *y*-axis. The total amount of glycerol added to the reactor, normalized by the actual volume of the reactor at every time is represented using the *y*-axis at the right side. The average result of two independent cultivations is shown. The error bars correspond to the standard deviation for the duplicate.

The use of FeedBeads® mimics the conditions at a fed-batch culture, where cells grow under substrate-limiting conditions, i.e., at growth rates below the µ_max_. The strains PpHP8 and PpHP18, which showed the highest 3-HP yields in FeedBeads® cultures, were thus further tested in a fed-batch bioreactor culture using a pre-programmed exponential glycerol feeding strategy to maintain the growth rate at 0.075 h^−1^, which is approximately half the value of the µ_max_ of the two strains (0.133 h^−1^ and 0.165 h^−1^, respectively). The feeding phase finished when the equivalent to 195 g L^−1^ of glycerol were added to the reactor, to compare the results with our previous experiments where the same overall amount of substrate was used (Fina et al., 2021). Thereafter, the cultivation was finalised when the DO signal increased, indicating the depletion of any residual glycerol that might have been accumulated during the late phase of the fed-batch culture. The cultivation profiles for both strains are shown in Figure 4.

At the end of the batch phase, PpHP8 produced 4.92 ± 0.17 g L^−1^ 3-HP, while the strain PpHP18 produced 4.11 ± 0.17 g L^−1^ 3-HP. These results agree with the observations from the mini bioreactors, where the yield of PpHP8 was higher than the yield of PpHP18. However, 3-HP concentration at the end of the fed-batch culture of PpHP18 reached 37.05 ± 0.73 g L^−1^, while the PpHP8 strain reached 35.40 ± 1.85 g L^−1^. Thus, the yield during the feeding phase was higher for PpHP18 than PpHP8. Moreover, the fact PpHP18 µ_max_ is higher than the one of PpHP8 resulted in a shorter batch phase (22 h compared to 24 h). Previous studies revealed that *P. pastoris* cannot sustain the growth rate in fed-batch cultivations at a pre-set µ of 0.10 h^−1^ when 3-HP concentrations reached concentrations above 15 g L^−1^, leading to the accumulation of glycerol and D-arabitol in the reactor medium (Fina et al., 2021). This phenomenon is still observed in PpHP8 strain growing at 0.075 h^−1^, where 17.41 g L^−1^ of glycerol and 6.01 g L^−1^ of D-arabitol accumulated into the reactor, while no glycerol nor D-arabitol accumulation were observed for PpHP18. For this reason, the PpHP18 cultivation was finalised just after stopping the addition of the feeding. Overall, the fed-batch for PpHP18 lasted 52 h, while the fed-batch of PpHP8 lasted 56.5 h. Thus, the volumetric productivity of PpHP18 was significantly higher than the volumetric productivity of PpHP8 (0.712 ± 0.010 g L^−1^ h^−1^ compared to 0.627 ± 0.033 g L^−1^ h^−1^). The overall product yield on glycerol was 0.194 ± 0.004 Cmol^−1^ for PpHP18 and 0.186 ± 0.010 Cmol^−1^ for PpHP8.

The productivity obtained for the strain PpHP18 is the highest productivity reported in yeast, and, to our knowledge, it is also the highest productivity reported using the malonyl-CoA pathway to produce 3-HP in any microorganism. Furthermore, 37.05 g L^−1^ is the highest 3-HP concentration reported in yeast, highlighting the potential of the combined use of the *P. pastoris* cell factory and glycerol as feedstock for 3-HP production. Notably, the metabolically engineered PpHP18 strain showed a 50% increase in the final 3-HP titre and yield, and a 31.9% increase in volumetric productivity compared to the initial strain (PpHP6).

## Conclusion

Production of 3-HP in *P. pastoris* through the malonyl-CoA reductase has been reported. The aim of this work was to improve its production by several metabolic engineering strategies. Improvement of the 3HP yield was obtained by adding a second copy of *mcr*-*C*_*Ca*_ . However, 1) increasing the availability of malonyl-CoA by overexpressing a post-translationally unregulated Acc1 from *P. pastoris;* 2) overexpressing endogenous acetyl-CoA pathway (ACS, Ald6, and Pdc1) or 3) limiting the production of arabitol by deleting *ArDH* did not improve 3-HP production in small-scale cultures. Nevertheless, deletion of *ArDH* improved the 3-HP production in fed-batch bioreactor cultures.

The work of this study demonstrates that the system and the mode of cultivation clearly affected the phenotype of the strains. For instance, the strain PpHP18 showed the highest production of 3-HP in fed-batch cultivation mode but not in deep-well plates and mini bioreactors operated in batch. This provides clear evidence on the importance to implement robust and reliable small-scale cultivation methods allowing for the mid/high throughput characterisation of strain performance under bioprocess-like conditions. However, the results obtained with the FeedBeads® were similar to the ones obtained in fed-bath cultures, thereby validating this methodology as a reliable method for strain screening/ranking and fast translation to bioreactor-scale.

Overall, our study showed that the multiplexed screening methodologies allowed to improve the information content, thereby supporting the formulation of hypotheses of previously unidentified metabolic bottlenecks such as that the supply of cytosolic acetyl-CoA may be limiting 3-HP production. Nonetheless, further studies, e.g., using ^13^C-based metabolic flux analysis and metabolomics are needed to design novel metabolic engineering strategies.

## Data Availability

The original contributions presented in the study are included in the article/Supplementary Material, further inquiries can be directed to the corresponding author.
