# Corrigendum: Combining metabolic engineering and multiplexed screening methods for 3-hydroxypropionic acid production in *Pichia pastoris*

**DOI:** 10.3389/fbioe.2022.1003012

**Published:** 2022-09-30

**Authors:** Albert Fina, Stephanie Heux, Joan Albiol, Pau Ferrer

**Affiliations:** ^1^ Department of Chemical, Biological and Environmental Engineering, Universitat Autònoma de Barcelona, Bellaterra (Cerdanyola del Vallès), Catalonia, Spain; ^2^ TBI, Université de Toulouse, CNRS, INRAE, INSA, Toulouse, France

**Keywords:** 3-hydroxypropionic acid, *Pichia pastoris*, glycerol, malonyl-CoA, acetyl-CoA, metabolic engineering

In the published article, there was an error in **Affiliation 1**. It should be “Department of Chemical, Biological and Environmental Engineering, Universitat Autònoma de Barcelona, Bellaterra (Cerdanyola del Vallès), Catalonia, Spain.”

There was a misspelling in the article title of the word “Hydroxyproprionic.” The correct title should be “Combining Metabolic Engineering and Multiplexed Screening Methods for 3-Hydroxypropionic Acid Production in *Pichia pastoris*.”

Several references were formatted incorrectly. Corrections have been made to the following **References**:

“Baumann, K., Dato, L., Graf, A. B., Frascotti, G., Dragosits, M., Porro, D., et al. (2011). The Impact of oxygen on the transcriptome of recombinant *S. cerevisiae* and *P. pastoris*—A comparative analysis. *BMC Genomics* 12, 218. doi: 10.1186/1471-2164-12-218.”

“Cámara, E., Albiol, J., and Ferrer, P. (2016). Droplet digital PCR-aided screening and characterization of *Pichia pastoris* multiple gene copy strains. *Biotechnol. Bioeng*. 113, 1542–1551. doi: 10.1002/bit.25916.”

“Cregg, J. M., Vedvick, T. S., and Raschke, W. C. (1993). Recent advances in the expression of foreign genes in *Pichia pastoris*. *Nat. Biotechnol.* 11, 905–910. doi: 10.1038/nbt0893-905.”

“Fang, H., Li, D., Kang, J., Jiang, P., Sun, J., and Zhang, D. (2018). Metabolic engineering of *Escherichia coli* for *de novo* biosynthesis of vitamin B12. *Nat. Commun.* 9, 4917. doi: 10.1038/s41467-018-07412-6.”

“Fina, A., Brêda, G. C., Pérez‐Trujillo, M., Freire, D. M. G., Almeida, R. V., Albiol, J., et al. (2021). Benchmarking recombinant *Pichia pastoris* for 3‐hydroxypropionic acid production from glycerol. *Microb. Biotechnol.* 14, 1671–1682. doi: 10.1111/1751-7915.13833.”

“Garcia-Ortega, X., Ferrer, P., Montesinos, J. L., and Valero, F. (2013). Fed-batch operational strategies for recombinant Fab production with *Pichia pastoris* using the constitutive GAP promoter. *Biochem. Eng.* J. 79, 172–181. doi: 10.1016/j.bej.2013.07.013.”

“Gassler, T., Heistinger, L., Mattanovich, D., Gasser, B., and Prielhofer, R. (2019). CRISPR/Cas9-mediated homology-directed genome editing in *Pichia pastoris*. *Methods Mol. Biol.* 1923, 211–225. doi: 10.1007/978-1-4939-9024-5_9.”

“Kildegaard, K. R., Jensen, N. B., Schneider, K., Czarnotta, E., Özdemir, E., Klein, T., et al. (2016). Engineering and systems-level analysis of *Saccharomyces cerevisiae* for production of 3-hydroxypropionic acid via malonyl-CoA reductase-dependent pathway. *Microb. Cell Fact.* 15, 53. doi: 10.1186/s12934-016-0451-5.”

“Kim, J. W., Ko, Y. S., Chae, T. U., and Lee, S. Y. (2020). High‐level production of 3‐hydroxypropionic acid from glycerol as a sole carbon source using metabolically engineered *Escherichia coli*. *Biotechnol. Bioeng.* 117, 2139–2152. doi: 10.1002/bit.27344.”

“Marx, H., Mecklenbräuker, A., Gasser, B., Sauer, M., and Mattanovich, D. (2009). Directed gene copy number amplification in *Pichia pastoris* by vector integration into the ribosomal DNA locus. *FEMS Yeast Res.* 9, 1260–1270. doi: 10.1111/j.1567-1364.2009.00561.x.”

“Maurer, M., Kühleitner, M., Gasser, B., and Mattanovich, D. (2006). Versatile modeling and optimization of fed batch processes for the production of secreted heterologous proteins with *Pichia pastoris*. *Microb. Cell Fact.* 5, 37. doi: 10.1186/1475-2859-5-37.”

“Peiro, C., Millard, P., de Simone, A., Cahoreau, E., Peyriga, L., Enjalbert, B., et al. (2019). Chemical and metabolic controls on dihydroxyacetone metabolism lead to suboptimal growth of *Escherichia coli*. *Appl. Environ. Microbiol.* 85, e00768–19. doi: 10.1128/AEM.00768-19.”

“Pereira, H., Azevedo, F., Domingues, L., and Johansson, B. (2022). Expression of *Yarrowia lipolytica* acetyl-coa carboxylase in *Saccharomyces cerevisiae* and its effect on *in-vivo* accumulation of malonyl-CoA. *Comput. Struct. Biotechnol.* J. 20, 779–787. doi: 10.1016/j.csbj.2022.01.020.”

“Prielhofer, R., Barrero, J. J., Steuer, S., Gassler, T., Zahrl, R., Baumann, K., et al. (2017). GoldenPiCS: A golden gate-derived modular cloning system for applied synthetic biology in the yeast *Pichia pastoris*. *BMC Syst. Biol.* 11, 123. doi: 10.1186/s12918-017-0492-3.”

“Qiao, K., Imam Abidi, S. H., Liu, H., Zhang, H., Chakraborty, S., Watson, N., et al. (2015). Engineering lipid overproduction in the oleaginous yeast *Yarrowia lipolytica*. *Metab. Eng.* 29, 56–65. doi: 10.1016/j.ymben.2015.02.005.”

“Shiba, Y., Paradise, E. M., Kirby, J., Ro, D.-K., and Keasling, J. D. (2007). Engineering of the pyruvate dehydrogenase bypass in *Saccharomyces cerevisiae* for high-level production of isoprenoids. *Metab. Eng.* 9, 160–168. doi: 10.1016/j.ymben.2006.10.005.”

“Takayama, S., Ozaki, A., Konishi, R., Otomo, C., Kishida, M., Hirata, Y., et al. (2018). Enhancing 3-hydroxypropionic acid production in combination with sugar supply engineering by cell surface-display and metabolic engineering of *Schizosaccharomyces pombe*. *Microb. Cell Fact.* 17, 176. doi: 10.1186/s12934-018-1025-5.”

“Melo, N. T. M., Pontes, G. C., Procópio, D. P., de Gois e Cunha, G. C., Eliodório, K. P., Costa Paes, H., et al. (2020). Evaluation of product distribution in chemostat and batch fermentation in lactic acid-producing *Komagataella phaffii* strains utilizing glycerol as substrate. *Microorganisms* 8, 781. doi: 10.3390/microorganisms8050781.”

“Wen, J., Tian, L., Xu, M., Zhou, X., Zhang, Y., and Cai, M. (2020). A synthetic malonyl-CoA metabolic Oscillator in *Komagataella phaffii*. *ACS Synth. Biol.* 9, 1059–1068. doi: 10.1021/acssynbio.9b00378.”

In some subsections of **Materials and Methods**, the liter units are given in lower case (l), e.g., ml and μl, whereas in the rest of the manuscript, a capital letter is used for liters (L). For consistency, the capital letter nomenclature (i.e. L) should be used throughout the manuscript.

A correction has been made to **Materials and Methods**, *“Copy Number Determination by Droplet PCR”*, paragraph 1. The corrected sentence appears below:

“Subsequently, the genomic DNA was diluted to a concentration of 1 ng μL^−1^”.

A correction has been made to **Materials and Methods**, *“Copy Number Determination by Droplet PCR”*, paragraph 2. The corrected sentence appears below:

“Second, a master mix of 22.5 μL was prepared with the forward primer at 0.4 μM, the reverse primer at 0.2 μM, and the restricted genomic DNA at 0.08 ng μL^−1^. Afterwards, the master mix was mixed with 22.5 μL of EvaGreen 2X master solution and was thoroughly mixed by vortexing.”

A correction has been made to **Materials and Methods**, *“24 Deep-Well Plates Screening”*, paragraph 1. The corrected sentence appears below:

“*P. pastoris* strains were inoculated into 50 mL falcon tubes containing 5 mL of YPG (1% yeast extract, 2% peptone and 1% v/v glycerol) supplemented with 100 μg mL^−1^ zeocin (InvivoGen, CA, United States).”

A correction has been made to **Materials and Methods**, *“24 Deep-Well Plates Screening”*, paragraph 1. The corrected sentence appears below:

“The cells were grown overnight at 30°C and 200 rpm in an incubator shaker Multitron Standard (Infors HT, Bottmingen, Switzerland) with a 2.5 cm orbit. 50 μL of overnight-grown cultures were used to inoculate each well of a 24 deep-well plate containing 2 mL of Buffered Minimal Glycerol (BMG) medium, containing 100 mM potassium phosphate buffer pH 6, 1.34% yeast nitrogen base (YNB), 1% v/v glycerol, and 0.4 mg L^−1^ biotin.”

A correction has been made to **Materials and Methods**, *“Small-Scale Screening in Falcon Tubes Using FeedBeads®”*, paragraph 1. The corrected sentence appears below:

“The inoculum was prepared following the same protocol described for the deep-well plates screenings. Afterwards, 50-mL falcon tubes were filled with 5 mL of Buffer Minimal medium (BM; 100 mM potassium phosphate buffer pH 6, 1.34% YNB and 0.4 mg L^−1^ biotin), supplemented with one Glycerol FeedBeads® (SMFB12001, Kuhner Shaker GmbH, Germany).”

A correction has been made to **Materials and Methods**, *“Small-Scale Screening in Falcon Tubes Using FeedBeads®”*, paragraph 1. The corrected sentence appears below:

“This FeedBead ®releases 40 mg of glycerol in 48 h. The cultures were inoculated with 50 μL of the overnight saturated cultures. The falcon tubes were incubated in an incubator shaker at 200 rpm and 30°C for 48 h. Each clone was tested in triplicate. A triplicate control was performed by adding one FeedBead ® to a falcon with 5 mL of BM medium. These controls were used to determine the actual release of glycerol under the tested conditions.”

A correction has been made to **Materials and Methods**, *“Mini Bioreactors Screening”*, paragraph 2. The corrected sentence appears below:

“The bioreactor medium contained 2.5 g L^−1^ glycerol, 1.8 g L^−1^ citric acid, 0.02 g L^−1^ CaCl_2_ · 2 H_2_O, 12.6 g L^−1^ (NH_4_)_2_HPO_4_, 0.5 g L^−1^ MgSO_4_ · 7 H_2_O, 0.9 g L^−1^ KCl, 50 μL antifoam Glanapon 2000 kz (Bussetti and Co., GmbH, Vienna, Austria), 0.4 mg L^−1^ biotin and 4.6 mL L^−1^ of PTM1 trace salts (Maurer et al., 2006).”

A correction has been made to **Materials and Methods**, *“Mini Bioreactors Screening”*, paragraph 2. The corrected sentence appears below:

“Each mini bioreactor was filled with 15 mL of medium. The pre-inoculum was prepared as described for the other two screening methods (deep-well plates and falcon tubes with FeedBeads®). The overnight-saturated cultures were used to inoculate 250 mL shake flasks with 25 mL of YPG at a starting OD_600_ of 0.5–1.5.”

A correction has been made to **Materials and Methods**, *“Mini Bioreactors Screening”*, paragraph 3. The corrected sentence appears below:

“The 250 μL samples for culture supernatant analysis were automatically placed on 96-well plates with a 0.45 μm pore size filter bottom.”

A correction has been made to **Materials and Methods**, *“Fed-Batch Cultures in Bioreactors”*, paragraph 1. The corrected sentence appears below:

“The starting volume of each 1.3 L reactor vessel was 400 mL”.

A correction has been made to **Materials and Methods**, *“Fed-Batch Cultures in Bioreactors”*, paragraph 2. The corrected sentence appears below:

“The feeding medium composition was 400 g L^−1^ glycerol, 10 g L^−1^ KCl, 6.45 g L^−1^ MgSO_4_ · 7 H_2_O, 0.35 g L^−1^ CaCl_2_ · 2 H_2_O, 0.2 mL L^−1^ antifoam Glanapon 2,000 kz, 1.2 mg L^−1^ biotin and 15 mL L^−1^ PTM1 trace salts.”

A correction has been made to **Materials and Methods**, *“Fed-Batch Cultures in Bioreactors”*, paragraph 4. The corrected sentence appears below:

“From 0.5 to 2 mL of culture were filtered through pre-weighted glass microfiber filters (APFF04700, Merck Millipore). The filters were then washed with 10 mL of distilled water with 9 g L^−1^ NaCl and dried overnight at 105°C. The filters containing the dry biomass were weighted to calculate the CDW.”

A correction has been made to **Materials and Methods**, *“NMR Analysis”*, paragraph 1. The corrected sentence appears below:

“Prior to the analyses, 180 μL of filtered culture supernatant samples were mixed with 20 μL of 10 mM TSP (3-(trimethylsilyl)-[2,2,3,3-^2^H_4_]-propionic acid sodium salt), which was used as an internal standard.”

The authors apologize for this error and state that this does not change the scientific conclusions of the article in any way. The original article has been updated.

