# Supplementary material for: Combining Metabolic Engineering and Multiplexed Screening Methods for 3-Hydroxypropionic Acid Production in Pichia pastoris
Source: Front Bioeng Biotechnol. 2022 Jul 22;10:942304. doi: 10.3389/fbioe.2022.942304 (PMC9354023; doi:10.3389/fbioe.2022.942304)
Supplement: Supplementary file 1 [file DataSheet1.docx]

Supplementary Material

# Molecular Biology protocols

All restriction enzymes and Q5 high-fidelity polymerase were purchased to (New England Biolabs, MA, USA).

The strains PpHP2 and PpHP6 had been previously generated in our lab. The list of plasmids used in this study are listed in the Supplementary Table 1.

When using the GoldenPiCS plasmids, its associated protocol for Golden Gate cloning was used (Prielhofer et al., 2017). The same name code is used to refer to the overhang sequences generated using the Golden Gate restriction enzymes BsaI and BbsI is used.

Supplementary Table 1. Plasmids list and description.

| **Plasmid name** | **Features / Use** | **Source** |
| --- | --- | --- |
| pBIZi_pGAP_MCR_C | To generate BB3eN_pGAP_mcrC_TDH3tt | (Fina et al., 2021) |
| BB3eN_14 |  | (Prielhofer et al., 2017);  Addgene #1000000133 |
| BB1_pGAP_12 |  | (Prielhofer et al., 2017);  Addgene #1000000133 |
| BB1_23 |  | (Prielhofer et al., 2017); Addgene #1000000133 |
| BB1_TDH3tt_34 |  | (Prielhofer et al., 2017);  Addgene #1000000133 |
| BB1_23_MCR_C |  | This study |
| BB3eN_pGAP_mcrC_TDH3tt | Expression cassette  pGAP_ *mcr-C_Ca__*TDH3tt.  Integration at site *ENO1* intergenic region. | This study |
| pK_pGAP_ACC1* | Expression cassette pGAP_*ACC1**_AOX1tt | (Liu et al., 2019);  Addgene #126740 |
| BB3cK_pGAP_23*_pLAT2_Cas9 | Plasmid carrying a clonsing site for the sgRNA and the hCas9 expressed under the control of pLAT2. | (Gassler et al., 2019); Addgene #1000000136 |
| BB3cK_pGAP_23*_pLAT2_Cas9_RGI2 | Transcription of sgRNA targeting a double strand break at the *RGI2* locus.  Expression of hCas9 under the control of pLAT2. | This study |
| BB3nK_AD |  | (Gassler et al., 2019); Addgene #1000000136 |
| BB3nK_ACS1_at_RGI2 | Expression cassette pTEF1_*acs_Se_^L641P^*_RPS3tt flanked with 1 kb homology sequences targeting at the *RGI2* locus. | This study |
| BB3nK_ACS1_ALD6_at_RGI2 | Expression cassette pTEF1_*acs_Se_^L641P^*_RPS3tt and pMDH3_*ALD6_Sc_*_TDH3tt flanked with 1 kb homology sequences targeting at the *RGI2* locus. | This study |
| BB3cK_pGAP_23*_pLAT2_Cas9_ArDH | Transcription of sgRNA targeting a double strand break at the ArDH locus.  Expression of hCas9 under the control of pLAT2 | This study |

To obtain the plasmid pBIZi_pGAP_MCR_C, first, the coding sequence of *mcr-C_Ca_* was amplified using primers adding the BsaI recognition sequence and the overhangs described in the GoldenPiCS protocol. The PCR amplicon was cloned into BB1_23 using BsaI. As a result, the plasmid BB1_23_MCR_C was obtained. Sequence integrity was checked by Sanger sequencing. Finally, BB3eN_pGAP_mcrC_TDH3tt was obtained using BB3eN_14, BB1_pGAP_12, BB1_23_MCR_C, and BB1_TDH3tt_34 and the Golden Gate protocol reported above with the restriction enzyme BbsI. The targeted integration locus was the intergenic region upstream of *ENO1*. The plasmid BB3eN_pGAP_mcrC_TDH3tt was linearized using PmeI and transformed into *P. pastoris* strains PpHP2 and PpHP6 to generate the strains PpHP7 and PpHP8, respectively.

PpHP9 was generated from linearizing the plasmid pK_pGAP_ACC1* with AvrII and transforming into PpHP7. The targeted integration locus was pGAP. The plasmid pK_pGAP_ACC1* contains two selection markers (geneticin resistance and histidine prototrophy). As PpHP7 was derived from the parental X-33 strain from Invitrogen (Thermo Fisher Scientific, MA, USA), which is prototroph for histidine, geneticin was used for the selection of clones having incorporated the plasmid.

Supplementary Table 2. Gene name and locus targetted for the double strand break during CrisPR-Cas9-based genetic engineering. The sgRNA sequence is shown. The PAM sequence is underlined.

| Locus | sgRNA sequence (PAM underlined) |
| --- | --- |
| RGI2 (PAS_chr1-1_0407) | TCTCAACGTATTTATATGGTCGG |
| ArDH (PAS_chr2-2_0019) | TGGATCACGCACAATCAAGATGG |

To generate the plasmids and the donor DNA required for CrisPR-Cas9, a previously described protocol was used (Gassler et al., 2019). Two single guide RNA (sgRNA) were designed targeting an intergenic region upstream of gene RGI2 and the coding region of D-arabitol dehydrogenase (ArDH). The sequence of the complementary sgRNA targeting at each genomic locus are listed in Supplementary Table 2.

The plasmids BB3nK_ACS1_at_RGI2 and BB3nK_ACS1_ALD6_at_RGI2 were build to obtain the donor DNA to knock-in the expression cassettes of the modified Acetyl-CoA Synthase from *Salmonella enterica* harbouring a mutation L614P (*acs_Se_**) and the aldehyde dehydrogenase from *Saccharomyces cerevisiae* (*ALD6_Sc_).* The sequence of the whole expression cassettes pTEF1_*acs_Se_^L641P^*_RPS3tt and pMDH3_*ALD6_Sc_*_TDH3tt were purchased to Integrated DNA Technologies (IA, USA) in a HiFi gBlock. The sequence of *acs_Se_^L641P^* and *ALD6_Sc_* were codon optimized for *P. pastoris* expression using the codon optimization tool of Integrated DNA Technologies, avoiding the introduction of BsaI and BbsI recognition sites. The coding sequences were flanked with the promoter (pTEF1 or pMDH3) and terminator (RPS3tt and TDH3tt) sequences found in the GoldenPiCS kit. BsaI recognition sequences resulting in overhangs B and E were added to the gBlock pTEF1_*acs_Se_^L641P^*_RPS3tt, while BsaI recognition sequences resulting in overhangs E and C were added to the gBlock pMDH3_*ALD6_Sc_*_TDH3tt. To build the plasmid BB3nK_ACS1_at_RGI2, high-fidelity PCR was performed using genomic DNA from *P. pastoris* X-33 and primer pairs RGI_5H_FW_A/RGI_5H_RV_B and RGI_3H_FW_E/RGI_5H_RV_D (see Supplementary Table 3). The first two primers generated a 763 bp amplicon with BsaI recognition sites at both ends generating overhangs A and B. The second pair or primers generated a 733 bp PCR product with BsaI recognition sites at both ends generating overhangs E and D. The two amplicons were mixed with BB3nK_AD and the gBlock pTEF1_*acs_Se_^L641P^*_RPS3tt. Using the Golden Gate protocol described for BsaI-HFv2, the plasmid BB3nK_ACS1_at_RGI2 was obtained.

To generate BB3nK_ACS1_ALD6_at_RGI2, the second primers pair was switched to RGI_3H_FW_C/ RGI_5H_RV_D to generate a PCR product of the same length, but having BsaI recognition sequences resulting in overhang sequences C and D. Performing Golden Gate using the two PCR products, the two described gBlocks, and BB3nK_AD, the plasmid BB3nK_ACS1_ALD6_at_RGI2 was obtained.

To obtain the strains PpHP11 and PpHP13, 1 µg of the circular plasmid BB3cK_pGAP_23*_pLAT2_Cas9_RGI2 was transformed into strains PpHP7 and PpHP8, respectively, together with 3 µg of the donor DNA containing the expression cassette pTEF1_*acs_Se_^L641P^*_RPS3tt flanked with RGI homology sequences at both ends. The donor DNA was obtained from excision from plasmid BB3nK_ACS1_at_RGI2 using BbsI.

Similarly, to obtain the strains PpHP12 and PpHP14, 1 µg of the circular plasmid BB3cK_pGAP_23*_pLAT2_Cas9_RGI2 was transformed into strains PpHP7 and PpHP8, respectively, together with 5 µg of the donor DNA containing the expression cassettes pTEF1_*acs_Se_^L641P^*_RPS3tt and pMDH3_*ALD6_Sc_*_TDH3tt flanked with RGI homology sequences at both ends. The donor DNA was obtained from excision from plasmid BB3nK_ACS1_ALD6_at_RGI2 using BbsI.

Supplementary Table 3. Primers list and sequence.

| **Primer name** | **Sequence** |
| --- | --- |
| RGI_5H_FW_A | TTTTCGCGGTCTCCGATCGAGGTTTACAAGCTGTGATGTTCC |
| RGI_5H_RV_B | TCCGGTGGTCTCCCCGGTGATGAACTGCCCGTCAAATTG |
| RGI_3H_FW_C | TTTTCGCGGTCTCAAATTGAAGTGGCTTCATAATTTCAGAACTC |
| RGI_3H_FW_E | TTTTCGCGGTCTCAGGAGTTGAAGTGGCTTCATAATTTCAGAACTC |
| RGI_5H_RV_D | TCCGGTGGTCTCCAGCTCGTTCGCTATATTATCATAGCCCAG |
| PDC_FW | ACCAAGCAAATAAACGCAAAGAGCAAC |
| PDC_RV | CTTAGCATAGTACAGAGTGGAAGCGG |
| ArDH_5H_FW | CATGAGAGACATATAACATTTTACAGAGCGG |
| ArDH_5H_RV | CAGCAACCGTCTTTGCTTGC |
| ArDH_5H_PDC_RV | GCTCTTTGCGTTTATTTGCTTGGTCAGCAACCGTCTTTGCTTGC |
| ArDH_3H_PDC_FW | CGCTTCCACTCTGTACTATGCTAAGGATCCGGCTCGTCCTCATCA |
| ArDH_3H_FW | GCAAGCAAAGACGGTTGCTGTGATCCGGCTCGTCCTCATCA |
| ArDH_3H_RV | AGACAGGCCTTATGGAGAACA |
| ArDH_check_in_FW | CATAGTTCCAAGCTTCAGATTGG |
| ArDH_check_in_RV | GTATGCGACTTGAGGTTGTGG |

To knock-out the main D-arabitol dehydrogenase encoding gene (*ArDH*), homology sequences upstream and downstream of the recognition site of the sgRNA were selected. The donor DNA results of the junction of the two aforementioned sequenced. Integration of such donor DNA would result in the excision of the whole *ArDH* expression cassette. Two high-fidelity PCR were performed using the genomic DNA of *P. pastoris* X-33 as a template and primer pairs ArDH_5H_FW/ArDH_5H_RV and ArDH_3H_FW/ArDH_3H_RV. The two amplicons (805 bp and 904 bp, respectively) were joined using a standard Overlap Extension PCR protocol to generate the donor DNA. The strains PpHP15 and PpHP17 were obtained from transforming 1 µg of the circular plasmid BB3cK_pGAP_23*_pLAT2_Cas9_ArDH and 1 µg of the donor DNA into the strains PpHP8 and PpHP13, respectively.

Finally, to knock-in a second copy of the Pyruvate Decarboxylase encoding gene (*PDC1*) while knocking-out *ArDH*, the whole expression cassette of *PDC1* was amplified using the Q5 high-fidelity polymerase primers pair PDC_FW and PDC_RV and the genomic DNA from *P. pastoris* X-33 as a template. An amplicon of 3149 bp was obtained. Afterwards, the flanking homologous sequences to the *ArDH* targeting sgRNA were amplified from the genomic DNA of *P. pastoris* X-33 with primer pairs ArDH_5H_FW/ArDH_5H_PDC_RV and ArDH_3H_PDC_FW/ArDH_3H_RV. To obtain the donor DNA, the two amplicons (829 bp and 908 bp, respectively) were joined with ArDH using a standard Overlap Extension PCR protocol, resuting in a PCR fragment of 4835 bp. The strains PpHP16 and PpHP18 were obtained from transforming 1 µg of the circular plasmid BB3cK_pGAP_23*_pLAT2_Cas9_ArDH and 4 µg of the donor DNA into the strains PpHP8 and PpHP13, respectively.

The whole *PDC1* expression cassette was amplified from *P. pastoris* strains PpHP15, PpHP16, PpHP17, and PpHP18 using the colony PCR protocol with Q5 high-fidelity polymerase described elsewhere (Gassler et al., 2019). The PCR products were Sanger sequenced to confirm the integrity of the sequence.

To check that the endogenous *ArDH* expression cassette did not reintegrate in a different locus, a colony PCR with primer pairs ArDH_check_in_FW/ArDH_check_in_RV was performed to strains PpHP8, PpHP15, PpHP16, PpHP17, and PpHP18. These primers amplify a 526 bp PCR product within the coding region of *ArDH*. PpHP8 was used as a positive control. No PCR product was obtained for strains PpHP15, PpHP16, PpHP17, and PpHP18, confirming the correct excision of *ArDH* in these strains.

# References

Fina, A., Brêda, G. C., Pérez-Trujillo, M., Freire, D. M. G., Almeida, R. V., Albiol, J., et al. (2021). Benchmarking recombinant *Pichia pastoris* for 3-hydroxypropionic acid production from glycerol. *Microbial Biotechnology* 14, 1671–1682. doi:10.1111/1751-7915.13833.

Gassler, T., Heistinger, L., Mattanovich, D., Gasser, B., and Prielhofer, R. (2019). “CRISPR/Cas9-mediated homology-directed genome editing in *Pichia pastoris*,” in *Methods in Molecular Biology* (Humana Press Inc.), 211–225. doi:10.1007/978-1-4939-9024-5_9.

Liu, Y., Bai, C., Liu, Q., Xu, Q., Qian, Z., Peng, Q., et al. (2019). Engineered ethanol-driven biosynthetic system for improving production of acetyl-CoA derived drugs in Crabtree-negative yeast. *Metabolic Engineering* 54, 275–284. doi:10.1016/j.ymben.2019.05.001.

Prielhofer, R., Barrero, J. J., Steuer, S., Gassler, T., Zahrl, R., Baumann, K., et al. (2017). GoldenPiCS: A Golden Gate-derived modular cloning system for applied synthetic biology in the yeast *Pichia pastoris*. *BMC Systems Biology* 11, 1–14. doi:10.1186/s12918-017-0492-3.
